# Better than DEET Repellent Compounds Derived from Coconut Oil

**DOI:** 10.1038/s41598-018-32373-7

**Published:** 2018-09-19

**Authors:** Junwei J. Zhu, Steven C. Cermak, James A. Kenar, Gary Brewer, Kenneth F. Haynes, Dave Boxler, Paul D. Baker, Desen Wang, Changlu Wang, Andrew Y. Li, Rui-de Xue, Yuan Shen, Fei Wang, Natasha M. Agramonte, Ulrich R. Bernier, Jaires G. de Oliveira Filho, Ligia M. F. Borges, Kristina Friesen, David B. Taylor

**Affiliations:** 1 U.S. Department of Agriculture, Agricultural Research Service, Agroecosystem Management Research Unit, University of Nebraska, Lincoln, Nebraska 68583 USA; 2 U.S. Department of Agriculture, Agricultural Research Service, National Center for Agricultural Utilization Research, Peoria, Illinois 61604 USA; 3 Department of Entomology, University of Nebraska, Lincoln, Nebraska 68583 USA; 4 Department of Entomology, University of Kentucky, Lexington, 40546 USA; 5 Key Laboratory of Bio-Pesticide Innovation and Application of Guangdong Province, Department of Entomology, College of Agriculture, South China Agricultural University, Guangzhou, 510642 Guangdong Province China; 6 Department of Entomology, Rutgers University, New Brunswick, 08901 USA; 7 U.S. Department of Agriculture, Agricultural Research Service, Invasive Insect Biocontrol and Behavior Laboratory, Beltsville Agricultural Research Center-West, Beltsville, Maryland 20705 USA; 8 Anastasia Mosquito Control District, 120 EOC Drive, St. Augustine, Florida 32092 USA; 9 U.S. Department of Agriculture, Agricultural Research Service, Center for Medical, Agricultural and Veterinary Entomology, Gainesville, Florida 32608 USA; 10 Departamento de Microbiologia, Imunologia, Parasitologia e Patologia, Instituto de Patologia Tropical e Saúde Pública, Universidade Federal de Goiás – UFG, Goiânia, GO Brazil

## Abstract

Hematophagous arthropods are capable of transmitting human and animal pathogens worldwide. Vector-borne diseases account for 17% of all infectious diseases resulting in 700,000 human deaths annually. Repellents are a primary tool for reducing the impact of biting arthropods on humans and animals. N,N-Diethyl-*meta*-toluamide (DEET), the most effective and long-lasting repellent currently available commercially, has long been considered the gold standard in insect repellents, but with reported human health issues, particularly for infants and pregnant women. In the present study, we report fatty acids derived from coconut oil which are novel, inexpensive and highly efficacious repellant compounds. These coconut fatty acids are active against a broad array of blood-sucking arthropods including biting flies, ticks, bed bugs and mosquitoes. The medium-chain length fatty acids from C_8:0_ to C_12:0_ were found to exhibit the predominant repellent activity. In laboratory bioassays, these fatty acids repelled biting flies and bed bugs for two weeks after application, and ticks for one week. Repellency was stronger and with longer residual activity than that of DEET. In addition, repellency was also found against mosquitoes. An aqueous starch-based formulation containing natural coconut fatty acids was also prepared and shown to protect pastured cattle from biting flies up to 96-hours in the hot summer, which, to our knowledge, is the longest protection provided by a natural repellent product studied to date.

## Introduction

It is well-known that insect bites can cause local or systemic effects that lead to infectious or inflammatory responses in human and animals. Many blood-sucking insects (primarily in mosquitoes) transmit many pathogens primarily plasmodium (malaria), viruses causing West Nile, Zika, yellow fever, and dengue in humans, in addition to equine infectious anemia, and African swine fever in animals^1,2^. Biting flies, such as stable flies (*Stomoxys calcitrans*) and horn flies (*Haematobia irritans*), have been reported to not only reduce the productivity of livestock, and also to transmit Lumpy Skin Disease and Rift Valley viruses mechanically^3–5^. Furthermore, another two blood-sucking arthropods often found in urban environments, ticks and bed bugs, have recently experienced resurgences for which ticks are vectors of many human and animal pathogens^6,7^.

The use of repellents has become one of the most efficient ways to prevent disease transmission and the discomfort associated with insect bites^8^. DEET (N,N-Diethyl-*meta*-toluamide), developed in 1944, is considered by many as the gold standard of insect repellents^9^. It was first used by the military during World War II, and subsequently commercialized in 1957^1,10^. Although DEET has been the most extensively used personal arthropod repellent for over six decades, it has been frequently associated with human health issues, particularly for infants and pregnant women^11,12^.

In contrast, natural products including plant essential oils have been used for their insecticidal and repellent properties for at least two millennia in ancient China, Egypt, and India^13–15^. Among them, citronella oil was the first successful plant-based insect repellent, but its effectiveness is relatively short^16^. Hundreds of studies reporting thousands of plant-derived materials exhibiting repellent and insecticidal properties have been reported in recent years^17^. However, nearly all plant-based repellents derived from plant essential oils have limited residual activity (<2–4 hours)^18^, primarily due to their high volatility. Although, the residual activity of a few plant-based essential oils can be extended up to 8 hours by the addition of a fixative such as vanillin^19^. DEET (>25%) provides up to 10 hours of protection against mosquitoes^20^. There is considerable interest in developing plant-based repellents with greater efficacy and extended residual activity due to increasing regulations and growing negative public perceptions against synthetic repellents and insecticides like DEET^15^.

In the present paper, we report that medium chain length fatty acids derived from coconut oil that provide strong repellency to four different types of insect vectors (mosquitoes, ticks, biting flies and bed bugs). To our knowledge, this is also the first report showing that the longevity and effectiveness of these natural repellent compounds better than the gold standard repellent, DEET against those blood-sucking insects.

## Results

### Coconut oil analyses

Coconut oil is a highly saturated triglyceride oil known for its rich lauric (C_12:0_) and myristic acid (C_14:0_) content. Accordingly, the fatty acid composition of coconut oil used in this study was determined after transesterification to the corresponding fatty acid methyl esters. Gas chromatography (GC) and gas chromatography/mass spectrometry (GC/MS) was used to identify the fatty acids contained in the oil and determine their percentages (Table 1). The oil contained a series of C_8:0_ to C_18:2_ fatty acids, whereby the medium chain fatty acids (C_8:0_ to C_12:0_) accounted for ~70% of total fatty acid profile. Lauric acid (C_12:0_) was the predominant fatty acid accounting for 53% of total fatty acids.

**Table 1:** Fatty acid composition of coconut oil.

	Relative amounts (%)
Caprylic acid (C_8:0_)	6.85 ± 0.03
Capric acid (C_10:0_)	7.33 ± 0.02
Lauric acid (C_12:0_)	52.68 ± 0.11
Myristic acid (C_14:0_)	17.14 ± 0.04
Palmitic acid (C_16:0_)	8.44 ± 0.03
Stearic acid (C_18:0_)	1.29 ± 0.01
Oleic acid (C_18:1_)	6.02 ± 0.10
Linoleic acid (C_18:2_)	0.34 ± 0.01

### Repellency of coconut fatty acids

#### Biting flies

**Figure 1:**
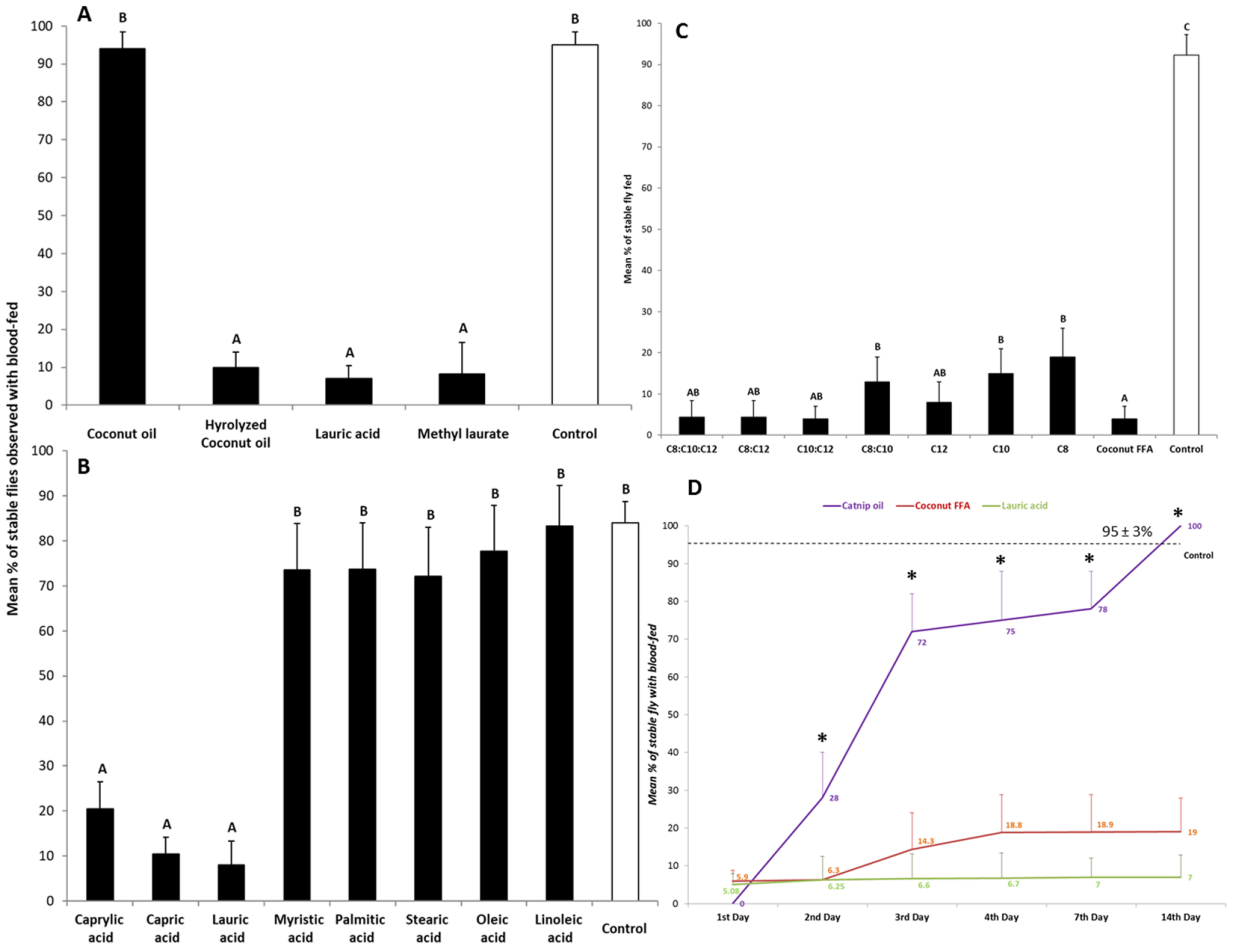
(**A**) Percentage of blood-feeding using 48-hr starved stable flies (*Stomoxy calcitrans*) with treatments of coconut oil, coconut fatty acids, lauric acid and its methyl ester observed in lab behavioral assays using modified K&D boxes; as well as those from treatments of all compositional acids. (**B**) Comparisons of percentiles of blood feeding of 48-hr starved stable flies while treated with different combinations of the compositional fatty acids from hydrolyzed coconut oil. (**C**) Different letters on top of bars indicate significant differences among treatments (ANOVA, followed by Scheffe tests, *P* < 0.05). Error bars show standard errors of the means. N = 22–40. (**D**) Comparisons of the longevity of mean percentages of repellency against stable fly blood-feeding observed in the modified K&D boxes from coconut fatty acids, lauric acid and catnip oil. *Indicates significant differences found among time periods after treatments (df = 5, 109, F = 28.2, P < 0.0001); Different letters on top of bars (same color) indicate time after treatments differ significantly at P < 0.0001, df = 2, 40–50, F = 12.3–68.5.

Bioassays using modified K&D module^21,22^ showed that coconut oil itself had little repellency against stable flies, *Stomoxy calcitrans* (Fig. 1A). In contrast, the coconut fatty acid mixture, lauric acid, and methyl laurate provided strong repellency against the stable flies. To examine if a specific fatty acid present in coconut oil had greater repellency, each coconut fatty acid was tested individually against the flies (Fig. 1B). Interestingly, we found only C_8:0_, C_10:0_ and C_12:0_ fatty acids exhibited high repellency levels against stable flies, with >90% repellency at a dose of 1 mg/cm^2^ (Fig. 1B; df = 5,244; F = 107.1 P < 0.0001). No significant differences were found in the least repellency concentrations between the coconut fatty acids and its neat major constituent, lauric acid, (Table 2). Figure 1C shows how binary and ternary blends of the active fatty acids can influence repellency. As shown, significant differences in repellency were observed between the binary and ternary blends of medium chain acids and their individual acids, except lauric acid (Fig. 1C; df = 8,217; F = 24.3, P < 0.0001). Repellency longevity tests showed a two-week effectiveness against stable flies found from both the coconut fatty acids and lauric acid at an application dosage of 20 mg (1 mg/cm^2^), whereas the positive control, catnip oil, only lasted for one day (Fig. 1D).

**Table 2:** Comparisons of the least repellency concentrations (LR_50_ and LR_90_ at mg/cm^2^) of coconut free fatty acids and its major constituent (lauric acid) against stable flies, *N* = 5.

	LR_50_ (95% C.I.)	LR_90_ (95%, C.I.)
Coconut fatty acids	3.98 (1.91–6.39)	13.90 (8.47–40.39)
Lauric acid	3.69 (3.23–6.53)	13.61 (9.11–32.59)

**Figure 2:**
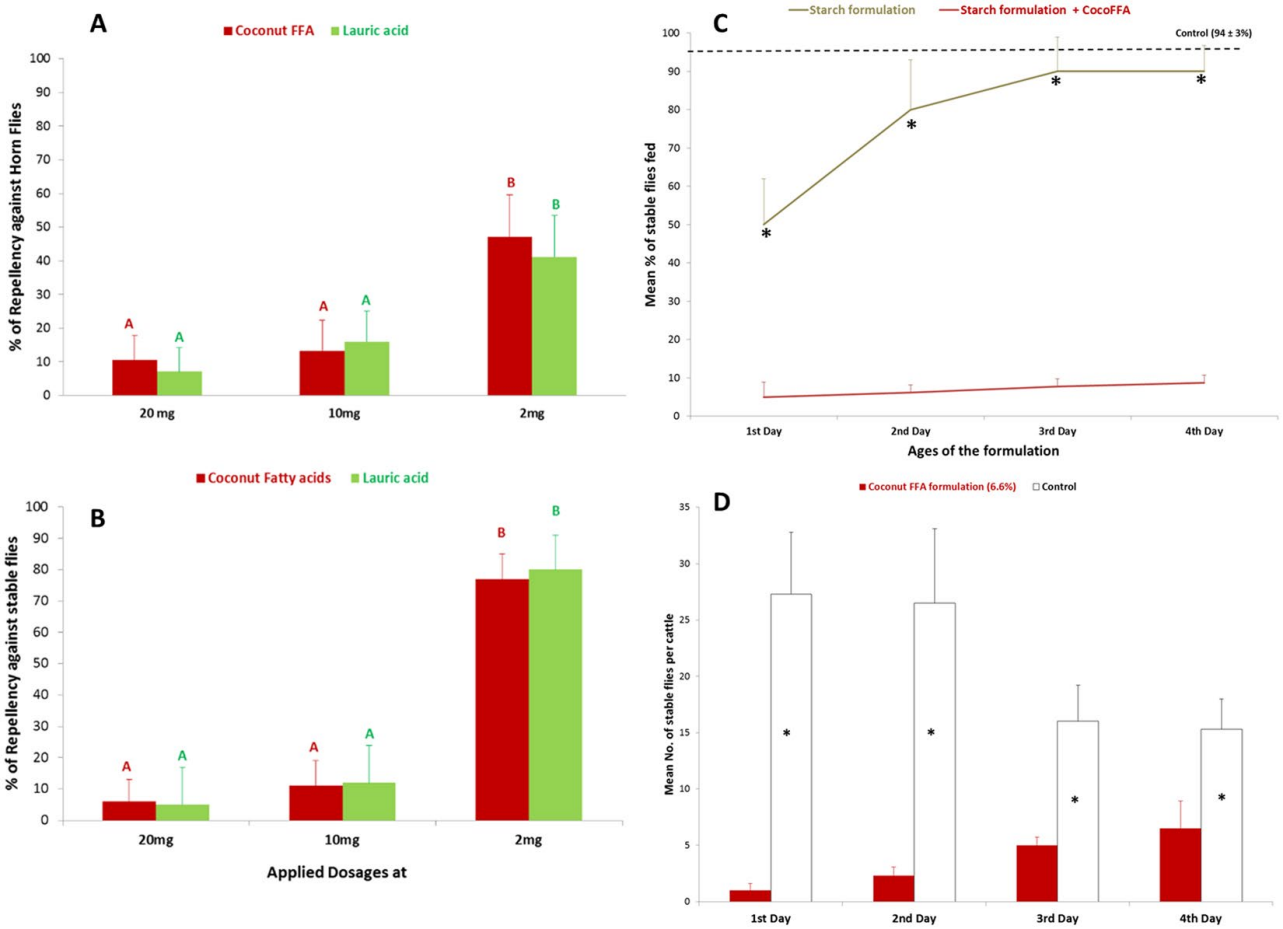
Dose response tests of repellency (as % of blood-fed) from coconut fatty acids and lauric acid against horn flies (**A**) and stable flies (**B**). Different letters on top of bars (same colors) indicate significant differences among three doses tested (ANOVA followed by Scheffe test, P < 0.05). N = 15–26. (**C**) Percentages of blood-feeding of 48-hr starved stable flies with treatments of starch based formulations with or without coconut fatty acids (CocoFFA). *Indicates significant differences found among time periods after treatments (T-test, P < 0.05); dashed line indicated mean % of blood feeding from controls. (**D**) Time-course of adult stable flies landing on legs of cattle treated with 6.6 wt.% coconut fatty acids in a starch-based formulation (starch formulation + coconut fatty acids) versus control treatment (starch formulation). *Indicates significant differences found among time periods after treatments (T-test, P < 0.01).

In addition to repellency against stable flies, the coconut fatty acids also effectively repelled another biting fly, *Haematobia irritans* (horn fly) (Fig. 2A). Dose-response tests demonstrated that the minimum effective concentration of repellency from coconut fatty acids was at 0.5 mg/cm^2^ against both biting flies (Fig. 2A,B; df = 2,48; F = 3.2–4.38, P < 0.05). In contrast, the average blood feeding from both flies in the control experiments were 92–98%. When the coconut fatty acids (CocoFFA) were formulated into an aqueous starch composite, the formulation showed over 90% feeding deterrence in the laboratory bioassays compared to a starch only formulation that showed less than 20% deterrence in 4 days (Fig. 2C; t = 2.12–2.48, P < 0.05). Furthermore, the starch containing the coconut fatty acids was shown to protect pastured cattle for up to 96 h against biting flies (Fig. 2D; t = 2.01–2.57, P < 0.05).

#### Bed bugs

**Figure 3:**
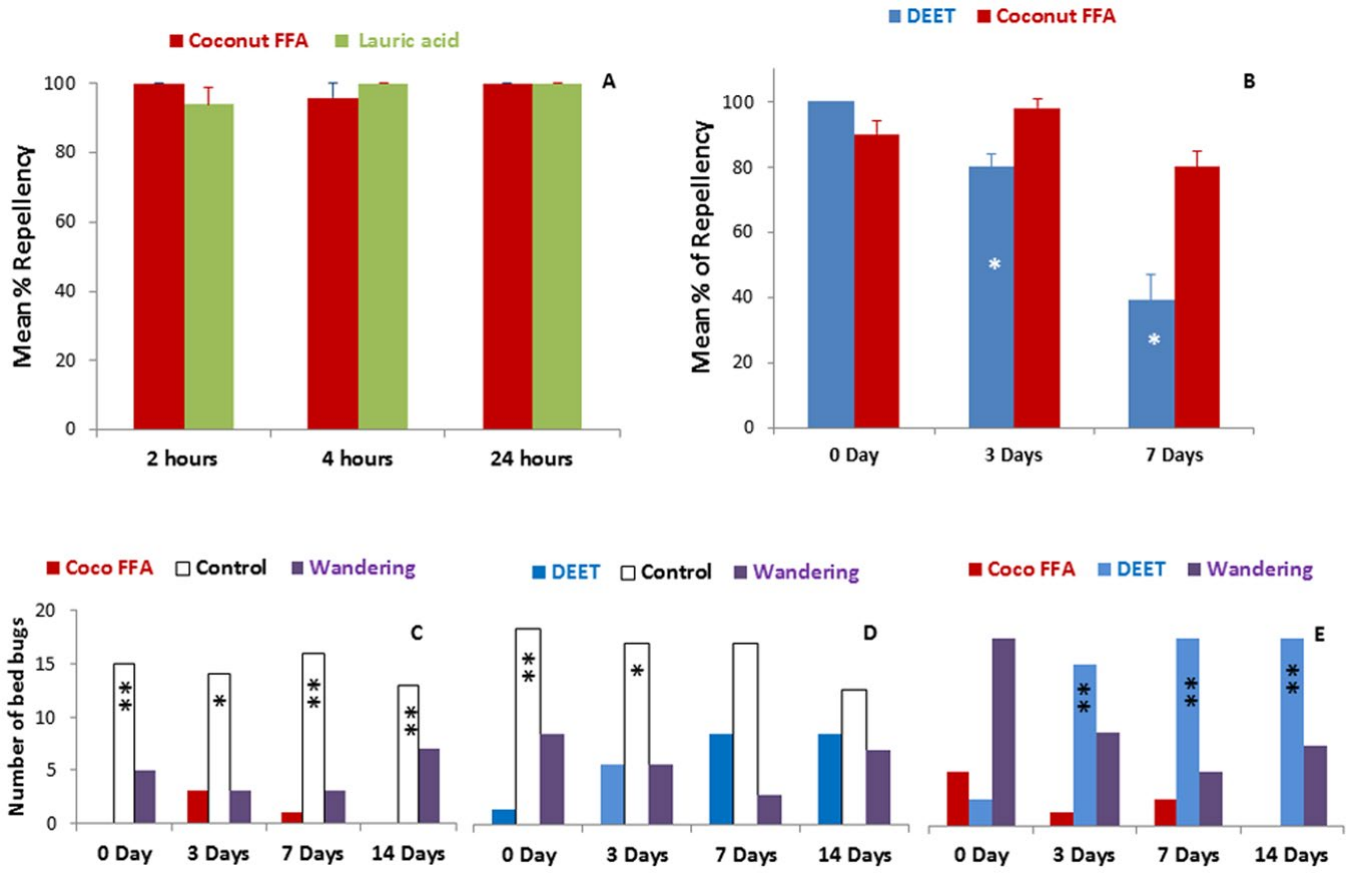
Comparisons of mean percentages of repellency from the coconut fatty acids, DEET and control against bed bugs. (**A**,**B**) Bars with an asterisk indicate significant differences, P < 0.05, Student’s T-test. Repellent tests with coconut oil fatty acids (coconut FFA) and DEET for bed bugs. (**C**) Bed bugs chose to rest on control tents (hexane-treated) when given a choice of coconut FFA -treated tent. This effect lasted on tents treated 14d earlier. (**D**) Initially bed bugs chose control tents over DEET-treated tents (0d and 3d), but this effect was no longer significant at 7d and 14d. (**E**) At 0d bed bugs did not discriminate between DEET and NT, preferring to wander in the test arena. However from 3d to 14d after tent treatment bed bugs chose to rest on DEET-treated over NT-treated tents. Binomial statistical tests were used with the null hypothesis that bed bugs would not discriminate between the two treatments (*P < 0.05; **P < 0.0.01). N = 12, 20 bed bugs per group.

Week-long repellency from coconut oil fatty acids to bed bugs, *Cimex letularius*, was demonstrated using two behavioral assays from two independent laboratories (Fig. 3). Results from the petri-dish assay showed that no significant differences in repellency was observed between coconut fatty acids and its major compound, lauric acid within a 24-hour period (Fig. 3A). While comparing the longevity of repellent efficiency between DEET and the coconut fatty acids, a significant stronger repellency was found from the coconut fatty acids even on the 7th day after application, with over 80% repellency remaining (Fig. 3B; P < 0.05). In contrast, the repellency of 10% DEET started to decrease on the third day after application. A second lab bioassay was designed to test bed bug choice between paired tents (harborages) treated with the coconut fatty acids, DEET and or a control showed an increase choice of DEET after 3 days, while the coconut fatty acids treated tents held strong repellency for up to 2 weeks (Fig. 3C–E, ^*^P < 0.05; ^**^P < 0.001).

#### Ticks

**Figure 4:**
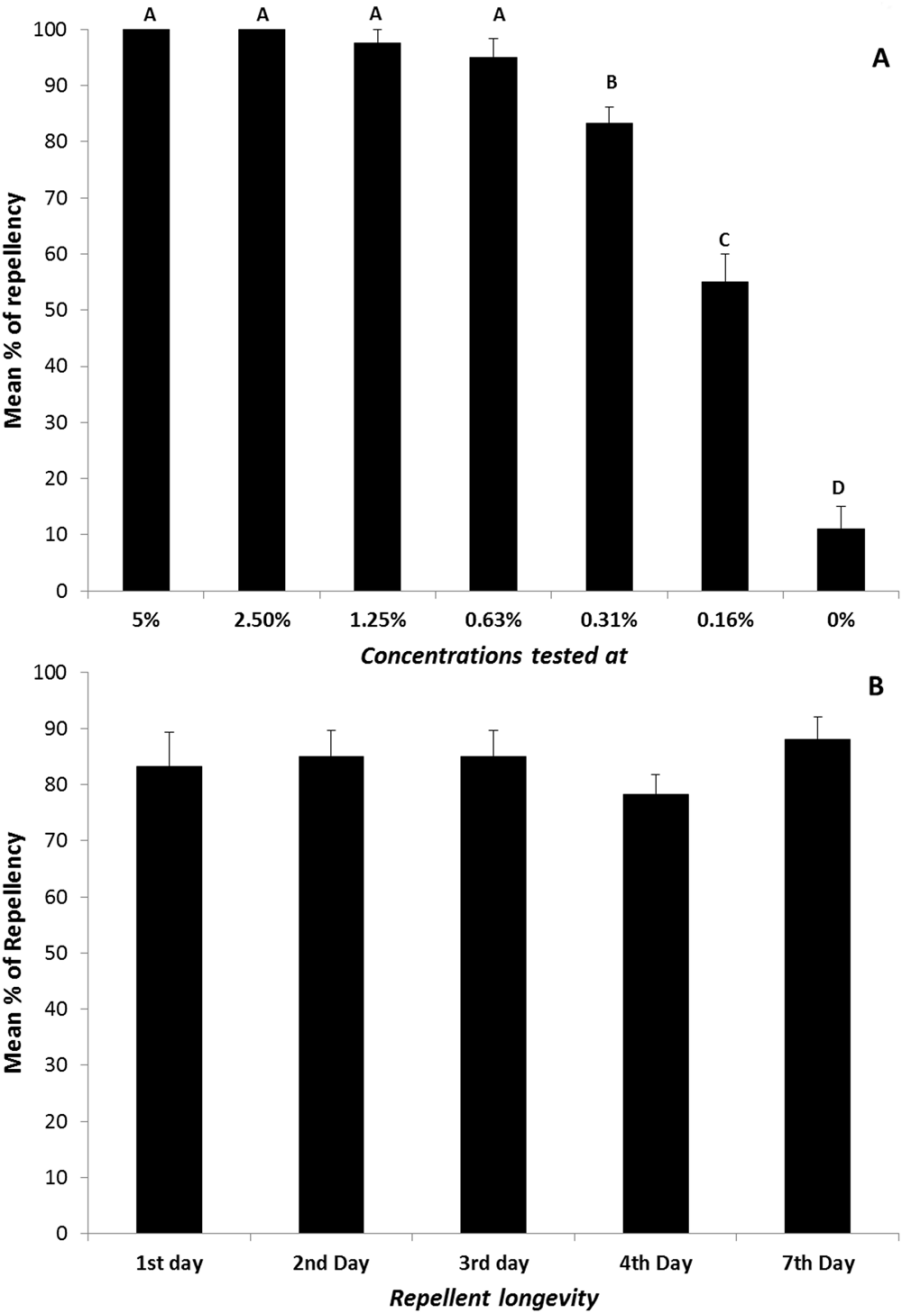
Dose responses of repellency and longevity tests from coconut fatty acids against two tick species, *A*. *americanum* (**A**); *R*. *sanguineus* (**B**). Different letters on top of bars (**A**) indicate significant differences among different concentrations tested (ANOVA followed by Scheffe test, P < 0.05). N = 4–5 for *A*. *americanum;* N = 10 for *R*. *sanguineus*.

The coconut fatty acids showed strong repellency to two tick species (Fig. 4A; df = 5,20; F = 4.71, P < 0.01). For the lone star tick, *Amblyomma americanum*, over 95% repellency was observed when test concentrations were above 0.625% (0.05 mg/cm^2^) in a veretical filter paper assay. A petri dish bioassay demonstrated that the coconut fatty acids provided protection for up to 7 days, and had a repellency between 84% and 88% to brown dog ticks, *Rhipicephalus sanguineus* (Fig. 4B).

#### Mosquitoes

When testing repellency of the coconut fatty acids on yellow fever mosquitoes, *Aedes aegypti*, a minimum required dose of 0.5 mg/cm^2^ was needed to effectively repel the mosquitoes (Table 3). A further test demonstrated that the coconut fatty acids at 25% (0.42 mg/cm^2^) provided over 93% of protection against yellow fever mosquitoes, while 73% of protection was observed from the same concentration containing its major compositional compound, lauric acid (Table 4).

**Table 3:** Minimum effective dosage (mg/cm^2^) of coconut free fatty acids required for biting protection against *Aedes aegypti*, N = 3–5.

	Dosage required
Coconut fatty acids	0.500 ± 0.125
Lauric acid	0.750 ± 0.000
DEET	0.047 ± 0.000

**Table 4:** Comparisons of biting protection (%) among different doses of coconut fatty acids, lauric acid and DEET against *Aedes aegypti* (arm-in-cage assay at 1^st^ hour, *N* = 3).

	6.25%	12.50%	25%	50%
Coconut fatty acids	67 ± 6	67 ± 6	93 ± 7	87 ± 6
Lauric acid	20 ± 0	20 ± 0	74 ± 6	60 ± 11
DEET	58–88^*^	77–97^*^	≈93^*^	not tested
Control	27 ± 7			

^*^Data extracted from references^32,33,42^.

### Comparisons of repellent efficacy between coconut oil fatty acids and DEET

**Figure 5:**
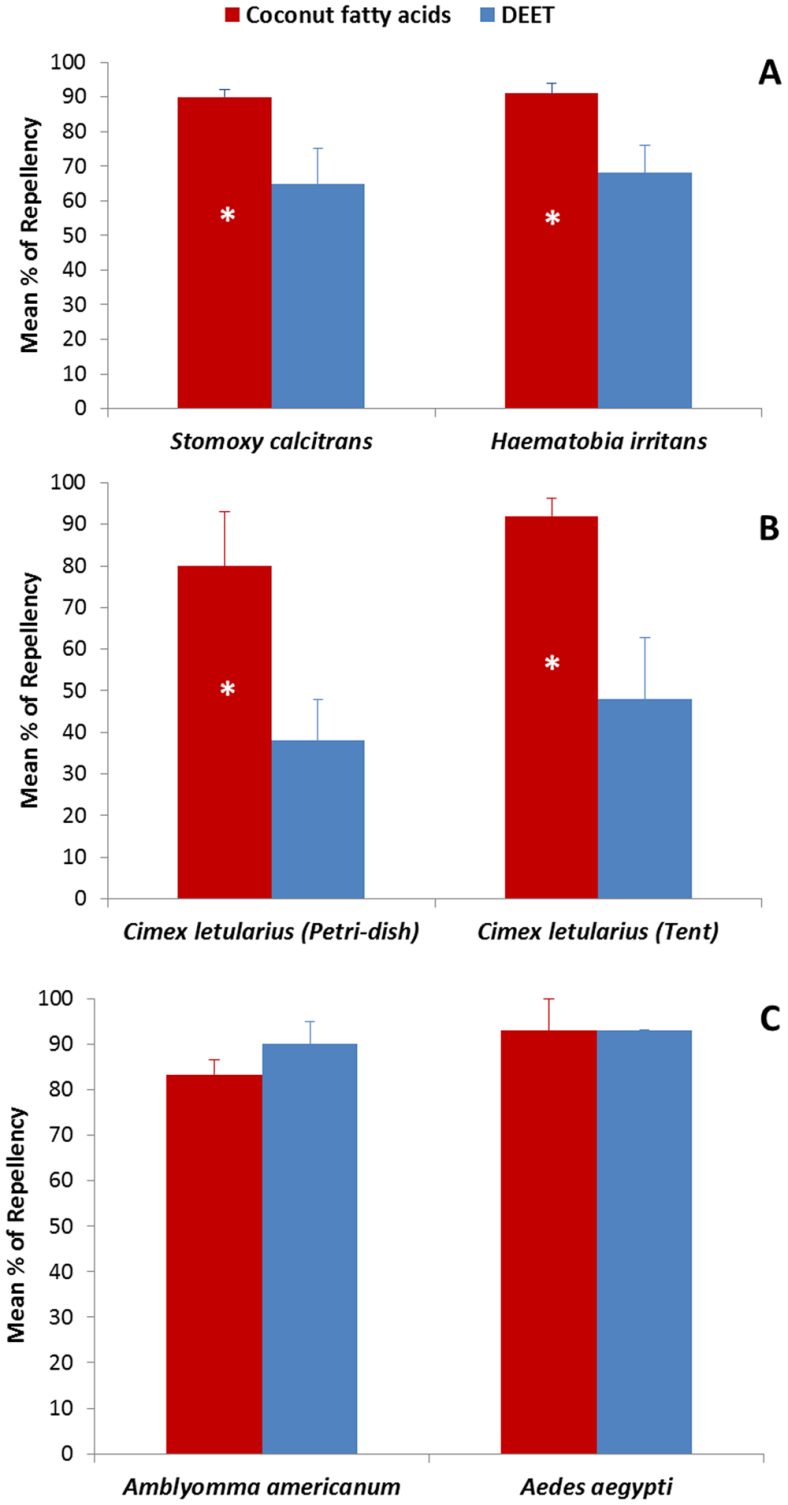
Comparisons of mean percentages of repellency between coconut fatty acids and DEET against biting flies (**A**), bed bugs (**B**), ticks and mosquitoes (**C**). An asterisk inside the bar indicates significant difference between the two treatments tested (P < 0.05, Student T-test). Error bars show standard errors of the means. *N* = 5–10 for A and B; N = 3–5 for C.

Strong repellency from the coconut fatty acids was demonstrated against three different types of blood-sucking insects (stable flies, horn flies and bed bugs), with levels of repellency that were better than DEET (Fig. 5A,B; P < 0.05). For lone star ticks, *Amblyomma americanum*, laboratory bioassays showed no significant differences in repellency between the coconut fatty acids and DEET when each was tested at concentration of 0.05 mg/cm^2^ (Fig. 5C). An equal repellency between the coconut fatty acids and DEET was found against yellow fever mosquitoes, *Aedes aegypti*, when each was tested at concentration of >0.4 mg/cm^2^ (Fig. 5C, Table 4, 25% of coconut fatty acids).

## Discussion

Coconut oil is an edible oil extracted from the kernel or meat of mature coconuts (*Cocos nucifera*) and is “generally recognized as safe” (GRAS)^23^. Of the fatty acids contained in hydrolyzed coconut oil, the C_8:0_, C_10:0_, and C_12:0_ fatty acids exhibited the highest levels of repellency against stable flies that have caused over 2 billion dollars in losses to the US livestock industry^24^. Similar levels of effectiveness to repel biting flies were observed between the coconut fatty acids and catnip oil at the first 24 hours. Catnip oil is considered as one of the strongest biting fly repellents identified thus far^22^, but the effectiveness decreased significantly after one day. Furthermore, methyl laurate, the methyl ester derived from lauric acid which is the main fatty acid present in coconut oil also exhibited toxicity (LT_90_ < 11 min) to the biting flies, while lauric acid itself was not toxic. Interestingly, coconut oil showed no repellency toward stable flies, possibly because the fatty acids are present as a larger triglyceride molecule^25^. Coconut oil’s lack of repellency suggests that the large bulky nature of the triglyceride structure may play a role in determining the repellency properties and not the presence of ester moieties since methyl laurate, which also contains an ester moiety similar to the triglyceride structure, is a highly effective repellent in addition to exhibiting toxicity. Higher repellent efficacy against stable flies was observed from mixtures of medium chain length acids rather than individual fatty acids and demonstrates a synergistic effect in repellency. More work in this area is needed to better understand the relationship between their chemical structures and repellency properties, particularly in stable fly olfactory and/or contact reception mechanisms as one of the major repellent compounds, lauric acid, is not overly volatile.

In addition to repellency against stable flies, the coconut fatty acids also repelled other blood-sucking insects including horn flies, bed bugs, brown dog ticks and lone star ticks. Ticks and horn flies are known to transmit many diseases in human and animals^26^. In recent years, the bed bug and ticks have also been reported as major public health concerns^27,28^. Our laboratory bioassays demonstrated stronger repellency by the coconut fatty acids against these insect vectors than DEET, which is considered the most effective repellent compound reported^29–32^. Although the coconut fatty acids exhibited strong repellency against biting flies, bed bugs and ticks, a relatively high concentration of the coconut fatty acids was required at the minimum effective dosage in comparison to DEET in order to prevent biting from yellow fever mosquitoes. However, in our study no significant differences in biting protection was observed between the coconut fatty acids and DEET at concentrations above 25%. Dodecanoic acid identified from tobacco smoke had previously been reported to repel yellow fever mosquitoes, but at >10 times higher concentration compared to DEET using an arm *in vivo* “cloth patch” assay^33^, which is similar to what we found from the current study. It is not clear why such a higher concentration of the coconut fatty acids (10 times higher than DEET) is required to repel mosquitoes effectively. Several excellent papers published regarding correlations between repellent chemical structures, olfactory receptors and their repellent efficacy suggest a complex interplay between these factors and the mechanisms involved^34–36^, and demonstrate the need to further understand how the medium chain length fatty acids function as repellents.

Medium chain length fatty acids, the major components of coconut oil, are readily available and inexpensive commodities that can also be obtained from other plant oils and animal fats^37^. The C8910 fatty acids (a commercial biopesticide) containing 1:1:1 mixture of synthetic octanoic, nonanoic and decanoic acids had been reported as repellents against several flies in laboratory trials^38^. Coconut fatty acids are considered non-toxic, and are widely used in the food and cosmetic industries, which related repellent products could also be developed to human to use in battling disease-transmitting mosquitoes^39^. By formulating the coconut fatty acids into an aqueous-based starch composite, an application is estimated to cost less than 0.1 US dollars per cattle. The initial testing on cattle demonstrated that the formulation can provide up to one week of protection against biting flies and ticks. It should be economically competitive to any currently available forms of biting fly control.

The present study represents the first report of a natural product repellant having more than one week of residual activity against biting flies, ticks, and bed bugs. In contrast, catnip oil, the best natural product repellent identified against biting flies so far, has less than 24-h of residual activity. Laboratory testing of an aqueous starch-based formulation containing 6.6 wt.% of coconut fatty acids prevented stable fly blood-feeding up to 7 days at room temperature (22–23 °C). While the same formulation of coconut fatty acid provided four days of protection under hot summer conditions (between 33–37 °C) via topical application on pastured cattle. However, a 20% formulation of catnip oil provided protection for a maximum of only 6 hours. To date, the longest residual activity of a plant based repellent, *Cymbopogan manti*, against mosquitoes provided less than 12 h of protection^18^.

Our laboratory behavioral assays have shown that the repellency from the coconut fatty acids against bed bugs is significantly strong. Bed bugs have the aggregation behavior, generally, they aggregate within refugia and returns to these harborages after each blood meal. In the bed bug Petri dish test, we introduced 5 nymphs and 5 males into the experimental arena simultaneously (Fig. 3B), whether the repellency efficacy of candidate compounds would be affected by the aggregation behavior of bed bugs was unclear. However, assays with individual bed bugs (tent assay) showed similar results to those with multiple bed bugs, indicating that the aggregation behavior did not counter or obscure these tests of repellency (Fig. 3C–E with individual bed bugs).

In conclusion, the present study has shown that fatty acids derived from coconut oil present stronger repellency against several blood-sucking insects, compared to the most commonly used repellent, DEET (3 out of 4 types). Over 90% of repellency against biting flies was demonstrated by coconut fatty acids and lauric acid, with two weeks of longevity in deterring blood feeding. Under field conditions, a low cost aqueous starch-based formulation of the coconut fatty acids provided up to 96 hours of protection against biting flies on cattle, which may be the longest lasting repellent of its type reported to date. This low-cost formulation can be adopted by livestock producers against biting flies. The GRAS status of these fatty acids should be easily accepted by public health professionals as preventative measures in battling mosquitoes, ticks and bed bugs, since these fatty acids have already been widely used in cosmetic industry^37^, thus potentially being safer to use as alternatives.

## Methods

### Oils, Chemicals and Repellents

Coconut oil was purchased from Swanson Health Products Inc. (Fargo, ND, USA). The coconut oil fatty acids were obtained from ACME HARDESTY (Blue Bell, PA, USA). Catnip essential oil was purchased from Bramble Berry Inc. (Bellingham, WA, USA). Fatty acids, methyl laurate, and DEET standards were purchased from Sigma-Aldrich (St. Louis, MO, USA), which all had a purity >98%. The tested chemicals were diluted to various concentrations using either ethanol or hexane that were also purchased from Sigma-Aldrich (99–100%). Genu pectin DD-slow set Z was obtained from CP Kelco (Atlanta, GA, USA). Waxy cornstarch (Waxy No. 1) was obtained from A.E. Staley Mfg. Co. (Decatur, IL, USA).

### Insects

Stable flies used for laboratory repellency tests were from colonies maintained at the United States Department of Agriculture (USDA), Agricultural Research Service (ARS), Agroecosystem Management Research Unit (Lincoln, NE, USA). The flies were maintained at 23 ± 2 °C with variable humidity (30–50% RH) and a light:dark photoperiod of 12:12 (L:D). Adult stable flies were fed with citrated bovine blood (3.7 g sodium citrate/liter) by soaking blood in a feminine napkin (Stayfree®, McNeil-PPC Inc., Skillman, NJ, USA) and placing it on top of the cage. Horn flies were shipped as pupae from an insecticide susceptible laboratory colony maintained at the USDA-ARS Knipling-Bushland US Livestock Insects Research Laboratory in Kerrville, TX, USA. Emerged horn fly adults were maintained under the same environmental conditions as stable flies, and fed in the same manner, with the exception that the blood-soaked pads were placed inside of their cages.

The bed bug strains were collected from human dwellings in Cincinnati, OH and New Jersey city, NJ, USA^40^. They were fed on defibrinated rabbit blood (Hemostat, Dixon, CA, USA) through a Parafilm™-membrane covered feeder which was heated to 39 °C with a circulating water bath. The bed bugs were maintained at 26°, 65 ± 5% RH, and a photoperiod of 14:10 (L:D) hr. Insects were evaluated 7–25d after emergence, and they had not been fed (for both nymphs and adults).

Unfed adult female and male lone star ticks were obtained from an *in vitro* colony at the USDA, ARS, Knipling-Bushland US Livestock Insects Research Laboratory. The colony was established from a Tick Rearing Facility at Oklahoma State University. All unfed adult ticks were maintained in an aquarium held at 27 ± 2 °C, 14:10 (L:D) photoperiod and sustained at 85% RH using a saturated salt solution. Engorged females of brown dog ticks were collected from naturally infested dogs in Goiânia, Goiás state, Brazil. A laboratory maintained tick colony was fed on rabbits (*Oryctolagus cuniculus*) using an apparatus glued in their backs. All free-living stages were maintained in a climatic chamber (27 °C and >80% RH). The ticks used in the experiments were aged between 7 and 21 days. The use of rabbits in this study was approved by the Committee on Ethical Animal Use of the Federal University of Goiás (CEUA/UFG, protocol number 024/2014). The care and use of the animals during this study were undertaken according to bioethics and animal welfare guidelines required by CEUA/UFG.

Mosquitoes used in this study were USDA strain *Aedes aegypti* reared in insectaries maintained at 26.6 °C, 85 ± 5% relative humidity (RH), and a photoperiod of 14:10 (L:D) h. Batches of 500 eggs were hatched in larval pans in 2.5 l of reverse osmosis water. Larvae were fed 1–3 g of liver and yeast mixture at a 3:2 ratio. Adult mosquitoes were supplied with 10% sucrose solution and a separate supply of reverse osmosis water.

### Repellency assays

#### Biting flies

The laboratory bioassay for testing repellent efficacy on fly biting/feeding consisted of a six-well feeding reservoir system similar to the K & D module^21,22^. Unlike assays used for mosquitoes, our bioassay with stable flies required no warm water. Adult flies were fed with blood once, then being starved, but water was provided 24–48 hours before the repellency test began. Stable flies were starved for 48 hours prior to testing, and 24 hours for horn flies. On the day of the test, small squares of the feminine napkin pad (3.75 × 4.75 cm) were cut to fit into wells of the module. When testing stable flies, the pads were soaked with ~5 mL of citrated bovine blood (local abattoir). The outer layers of the feminine napkin pads were cut and used for coating repellent candidates, which was made of 2 layers of 100% cotton flannel and a layer of ultra-thin nylon. Repellent candidates measured at three dosages, 2 mg, 10 mg and 20 mg, respectively, were dissolved in 300 µL of hexane (Burdick & Jackson High Purity Solvent, Muskegon, MI, USA), and then topically applied onto the outer layer evenly (4 × 5 cm). After air drying, it was placed on top of the blood-soaked pad. Approximately 3–5 starved flies were collected from the fly cages and transferred into each testing cell. After 4 hours, tested stable flies (anesthetized with CO_2_) were checked for feeding status by rupturing their abdomen to determine the presence of blood after the trials. Repellent assays were conducted daily at room temperature for at least 4 hours. Flies in the repellent bioassay were exposed to randomized treatments (different repellents and dosages), and repeated until at least 10 replicates were completed. Percentiles of repellency [(number of flies fed on control − number of flies fed in treatment)/number of flies fed on control × 100] was determined and transformed to arcsine square-root values for analyses of variance (ANOVA). Significant differences at P < 0.05 (SAS version 10; SAS Institute, Cary, NC, USA) were determined by analyses performed on the Least-Square Means due to the unequal number of observations among the treatments. Replicate numbers were determined by the number of treatments tested per day, and controls were always run simultaneously.

Dose-response repellent tests of coconut fatty acids and lauric acid against stable flies and horn flies were conducted using three different dosages described above. Hexane was used as the control. Results were analyzed as described above. The comparative study of using different ratios of the major repellent acids (C_8:0_, C_10:0_ and C_12:0_) was also conducted using the same procedures as described above, but only tested at the 20 mg dosage. The relative ratios of binary and three-component acid blends were based on the GC analyses of coconut oil fatty acids. These repellent bioassays were repeated for at least 6 replicates.

The longevity tests using coconut fatty acids and its starch-based formulation were carried out under laboratory conditions using the same repellent bioassay described above. Repellent layers loaded with coconut fatty acids or starch formulations (20 mg) were prepared inside a laboratory ventilation hood, and aged by hanging from a metal rack (1 m long) with metal clamps until all aged repellent layers were produced (1^st^ to 4^th^ day) that were run simultaneously with a total of 5 replicates of each treatments (at ages of 1^st^ to 4^th^ day-old plus controls, a positive control catnip oil was also tested to make sure the assay worked properly).

The least repellency concentration tests (LR_50_ and LR_90_) using coconut fatty acids and lauric acid against stable flies were carried out using various dosages (0.2 mg, 2 mg, 10 mg, and 20 mg). The bioassay was conducted using the same procedures described in the blood-feeding assay (modified K&D module). Hexane was used as the control. The experiment was repeated at least 5 times. A POLO PC program was used for Probit analysis of concentration-repellency data.

#### Bed bugs

Petri Dish Assay was used to quickly evaluate the comparative repellency of the repellent candidates and DEET (Rutgers University tests)^29^. Plastic Petri dishes (11.4 cm diameter by 3.8 cm height) were used as experimental arenas. For each arena, the inner wall coated with a thin film of fluoropolymer resin, bottom covered with a piece of filter paper. Filter paper was cut into two equal parts, one half was treated with a repellent using a Potter spray tower at 2.16 mg/cm^2^ of ethanol solution, the other half was sprayed with equal volume of 95% ethanol. A small piece of filter paper also was treated with the same repellent and folded into a tent shape with the treated surface facing down. The paper tent was placed on the repellent treated side, and the dish was uncovered. For the control treatment, one half of the filter paper and the tent was treated with 95% ethanol, the other half was untreated. 95% ethanol was used as solvent. 10% DEET (v/v), and 10% coconut fatty acids (m/m) were used to evaluate their repellency against bed bugs. All filter papers were treated on the same day, each kind of treated papers (95% ethanol-treated, 10% DEET-treated, or 10% coconut fatty acids-treated) were divided into three groups. They were kept in our laboratory (25 ± 1 °C, 20% relative humidity (RH)) for 0, 3, and 7days before experiment. The repellency of chemicals against bed bugs was tested at 0, 3, 7 days after application. Each filter paper was used only once. Five nymphs (fourth-fifth instar) and five males (age was unknown) were released in the center of each dish, the number of bed bugs on each side of the dish was recorded after 24 hours. All treatments were tested simultaneously. Each treatment was replicated 5–8 times. The assays were started between 3–4 hours into the dark cycle. Experiments were conducted in a walk-in chamber at 25 ± 1 °C, 20% RH, with a photoperiod of 12:12 (L:D). Repellency indices were calculated according to the formula: Repellency index = (C − T)/C × 100, where C = the mean number of bed bugs on the treated filter paper halves in all control dishes, and T = the number of bed bugs on the treated half of the filter paper in one test dish^29,41^. Repellency indices between the two compounds were compared using independent-samples T-test. (IBM SPSS Statistics 22.0, IBM, Armonk, NY, USA).

Behavioral responses of bed bugs were also tested using an indoor arena bioassay by another laboratory group (University of Kentucky test). The test materials were carried out in 9.5 cm inner diameter arena (ClimbUp Insect Interceptor™, Memphis, TN, USA). The inner well of this arena was covered with white filter paper (9.0 cm diam. Whatman, No. 2) that was fixed in place with double-sided tape to prevent bed bugs from getting beneath it. Test materials consisted of 20 µl of a 10% (w/w) hexane solution of DEET, coconut fatty acids, or hexane only. Test materials were applied to a 1.75 × 1.5 cm piece of filter paper that was pleated along the shorter midline to form a tent. The hexane was allowed to evaporate for 1 h before two tents were placed into the arena. Three different choice experiments were conducted: 1) Coconut fatty acids versus control; 2) DEET versus control; and 3) coconut fatty acids versus DEET. These test arenas were positioned in a wind tunnel so that different treatments were located across the wind line, and thus different treatments were isolated from each other. Arenas were placed on three levels of wire mesh shelf that was 0.7 m wide, 0.25 m deep, with 0.3 m between levels. The wind tunnel was 1 m wide by 0.9 m high by 2.4 m in length. The shelf and thus the arenas were placed only at the downwind end of the tunnel. The wind speed in the tunnel was 0.3 m/s with air evacuated from the laboratory through a fume hood. Tents were held in a separate fume hood for 0, 3, 7, and 14 days before being introduced into an arena. At about 9 h into the photophase, each bed bug was placed at the center point to the arena; equidistant from each tent. Room temperature during the choice test remained at 24 ± 2 °C. The position of bed bugs was noted at 16 h after release, which was 1 h after the initiation of the second photophase of the test. A total of 20 bed bugs were released with each treatment combination (at 0 d, 3 d, 7 d, and 14 d). Neither bed bugs nor tents were reused. Thus, a total of 240 insects were evaluated. The number of responses was analyzed by a binomial test using the null hypothesis that the two tents were chosen with equal probability. Insects that did not make a choice between the two tents (i.e., were wandering in the arena) were not included in the statistical analysis, but are shown in the figures.

#### Ticks

Repellency against nymphs of the lone star ticks was determined by using the vertical paper assay described previously^30^. A 4 × 7 cm rectangle of Whatman No. 4 filter paper was prepared by treating the central 4 × 5 cm zone with a volume of 165 µL of test solution. After drying, the paper strip was suspended from a bulldog clip hung from a holder. Ten lone star tick nymphs were released from a glass vial on the lower untreated end of the paper strip. Locations of the nymphs were recorded at 1, 3, 5, 10 and 15 min. Ticks were considered repelled if they stayed on the lower untreated zone or fell off the filter paper without having crossed into the upper untreated zone. Each treatment/concentration included three replicates.

For brown dog ticks, petri-dish bioassays were performed under controlled environmental conditions (at 27 °C and 70% RH) in complete darkness, based on the methodology described by Bissinger *et al*.^31^. The coconut fatty acid-treated filter papers were dried for 10 min under a fume hood prior to use in the assays. Six ticks (three males and three females) were placed in each arena along the line formed by the junction of treated and untreated papers. Control assays were made using clean paper versus clean paper. The positions of the ticks were evaluated at 24 h, 48 h, 72 h, 96 h and 1 week after the beginning of each experiment. Each experiment was replicated 10 times, with new ticks, for each individual compound.

In the Petri-dish bioassay repellency rates were determinate as the mean percentage ticks located on the untreated side of the Petri dish. The chi-square test was used for comparison of the tick choices, taking the significance level to be p < 0.05. When a higher significant proportion of ticks were found in the control side, the compound/concentration was considered as repellent.

#### Mosquitoes

Repellency was determined as the minimum effective dosage (MED, the minimum threshold surface concentration necessary to prevent mosquitoes from biting through the treated surface) of the coconut oil fatty acids to prevent bites through the fabric was first carried out at the USDA-ARS laboratory in Gainesville, FL. A 0.15 g sample of coconut oil fatty acids and DEET standard were added to prepare in 2 ml of solvent (acetone). Serial dilutions of the fatty acids and DEET were performed and each dilution was held in a separate vial. A 50 cm^2^ section of muslin cloth was added to each vial. Each volunteer wore each treated cloth to pinpoint the cloth which was treated with a concentration that failed (greater than or equal to 5 bites in one minute) and was next to an adjacent higher concentration passed (less than 5 bites in one minute). The lowest concentration passed was the MED for that test subject. Additional details on the bioassay methodology can be found in Carroll *et al*.^32^. There were three human volunteers in this study and all three provided written informed consent to participate in this study as part of a protocol (636–2005) approved by the University of Florida Human Use Institutional Review Board (IRB-01). A second repellent bioassay was conducted at Anastasia Mosquito Control Station in Florida. The three human volunteers aged from 30–60 involved in the second bioassay also followed the similar protocol approved by the Florida Human Use Institutional Review Board. An informed consent document was introduced to each individual volunteer in this study and received their approval for participation. Repellent treatments consisted of 1.0 mL of coconut fatty acids, lauric acid and DEET (control was also included), which was pipetted onto the forearms of the volunteers and applied from the wrist to the elbow by a gloved person to ensure full coverage of repellent. Approximately every 30 min from the start of the experiment each volunteer held their arm in a mosquito cage for 3 min. Protection failure was indicated by 2 mosquitoes landing and probing for more than 3 min on the treated area of the volunteer’s arm. Control volunteers were rotated with the other volunteers of the repellency test for 10 secs to 1 min to ensure that the mosquitoes still demonstrated attraction to hosts. The experiment was concluded after 6 h and 48 min when the last volunteer was probed for more than 3 min by 2 or more mosquitoes.

All methods related to mosquito repellency studies related to human participants from two laboratories were performed in accordance with the relevant guidelines and regulations approved by each institute.

### Chemical analyses of coconut fatty acids

The fatty acids from the coconut oil and coconut free fatty acids were identified by Agilent gas chromatography (GC) as well as with a GC combined with mass spectrometry (GC/MS) to confirm the identification of the acids. A 30-m FFAP column (0.25 mm i.d., 0.25 µm df (Sigma-Aldrich Inc.)) was used. Helium was used as the carrier gas. For analyzing the relative ratios of all acids, an Agilent GC system (6890 N) equipped with an FID detector and SP-2380 column (30 m × 0.25 mm i.d.). Parameters for SP-2380 analysis were: column flow 1.0 ml/min with a helium head pressure of 136 kPa; split ratio 50:1; programmed ramp 120 to 135 °C at 20 °C/min, 135 to 265 °C at 7 °C/min, hold 5 min at 265 °C; injector and detector temperatures set at 250 °C. For structure confirmation using GC-MS, the same temperature program was used as those of GC system. Saturated C_8_ − C_30_ FAME provided standards used to make FAME assignments. The FID results were standardized for the individual fatty acids and reported as w/w%. Relative proportions of free fatty acids from the coconut fatty acids were determined by acid methanolysis. Coconut fatty acids samples for GC were prepared by heating a 10 mg sample of coconut oil in 0.5 ml of 0.5 M KOH/MeOH to reflux on a heating block for 60 min in a sealed vial. After cooling to room temperature, 2 ml of 1 M H_2_SO_4_/MeOH was added to the vial, and the vial was resealed and heated to reflux on a heating block for 15 min. The solution was cooled and transferred to a small separatory funnel with hexane (1 mL) and washed with water (2 mL), dried over sodium sulfate, gravity filtered, placed in a GC vial with hexanes, sealed, and injected onto the GC.

### Repellent efficacy and longevity of coconut fatty acid formulation against biting flies on pasture cattle

A starch coconut fatty acid composite was prepared in a 4-L stainless steel Waring blender (Dynamics Corporation of America, New Hartford, CT, USA). A mixture of hot (80–90 °C) deionized water (1500 mL) and coconut fatty acids (152.0 g) was stirred to crudely emulsify the mixture. To the hot slurry, a mixture of waxy starch (200.5 g; moisture content 8.70%) and pectin (3.99 g; moisture content 12.09%) was added and stirred vigorously. The resulting slurry was delivered to the jet cooker utilizing a Moyno progressing cavity pump (Robbins Meyers, Springfield, OH, USA) at a flowrate of 1 l/min. The slurried mixture and steam were combined in a Penick and Ford hydroheater (Penford Corp, Cedar Rapids, IA, USA). Cooking temperature was 140 °C using steam supplied at 448 kPa, and the hydroheater backpressure set at 275 kPa. Approximately 2100 ml of a white opaque aqueous starch-coconut fatty acid mixture was collected and then cooled to room temperature while stirring (solids content ranged between 14–16%, as determined by freeze-drying accurately weighed amounts of the solution in duplicate). The solids content varied between experiments due to dilution of the cooked dispersion with variable amounts of condensed steam. The final composite had a solids content of 14.20% and was composed of 45.0% coconut fatty acids, 55.0% starch, and 85.80% water. The actual amount of coconut fatty acids contained in the formulation was 14.6% × 0.45 = 6.6 wt.%. The starch encapsulated coconut fatty acid composite was warmed in hot water bath and stirred well before the application. The aqueous starch-coconut fatty acid composite was stored at room temperature and subsequently brought to the field before being r topical applied on cattle.

The repellency against biting flies of the starch coconut fatty acid composite was tested on heifers under field conditions during the summer of 2017. The repellency tests were carried out in North Platte (University of Nebraska, West Central Research and Extension Center), NE, USA. Tests were conducted using criteria specified by the American Society for Testing and Materials (ASTM, 1980) and protocols approved by the Institutional Animal Care and Use Committee of the University of Nebraska (IACUC protocol no. 06–12–053 C). To test the effectiveness of the coconut fatty acid-based formulation, we used 12 heifers randomly assigned to two groups of six. Each cattle were an ear-tag number. Around 500 ml of the formulation was topically applied onto the whole-body surface of each cattle (including 4 legs as well). Since starch based formulation without coconut fatty acids added showed no repellency against biting flies, the control group cattle were not treated to reduce animal stress caused by treatment. Testing was done in two separate pastures of equivalent carrying capacity ranging in size from 10 to 17 hectares. Pastures were randomly assigned to each treatment that will enable precise estimates of treatment and treatment by period effects. Battery driven Fimco® sprayers were calibrated and used to make the application to the legs and belly of each animal. Cattle were individually restrained using a cattle chute during spray application and then released into the test pasture for exposure to ambient biting fly populations. The total number of biting flies on all four legs and belly of each cow were counted and expressed as the total number of stable flies per animal. Counts will be made between 1300 and 1600 during predetermined intervals by the same individual. The counts started from day 1 through day 4 (starting from Tuesday till Friday each week). These counts were confirmed using Microsoft Image Viewer from a window-based computer to examine photographs taken from a Nikon digital camera (D60) during the observations. Comparisons between treated and control animals for numbers of flies observed were performed using Student t-test. Results with P < 0.05 were considered statistically significant.

All methods related to biting fly repellency studies involved cattle from university of Nebraska North Platte research center were performed in accordance with the relevant guidelines and regulations approved by UNL animal committee.
